# Multiplatform Radiosurgery for Intracranial Meningiomas and Dose to the Dural Tail

**DOI:** 10.7759/cureus.12683

**Published:** 2021-01-13

**Authors:** Eduardo E Lovo, Alejandra Moreira, Paula A Navarro, Kaory C Barahona, Fidel Campos, Victor Caceros, Alejandro Blanco, Julio Arguello-Méndez, Leonor Arce, William O Contreras

**Affiliations:** 1 Radiosurgery, International Cancer Center, Diagnostic Hospital, San Salvador, SLV; 2 Functional Neurosurgery, Clínica Foscal Internacional, Bucaramanga, COL; 3 Radiosurgery, Robotic Radiosurgery Center, International Cancer Center Group, San José, CRI; 4 Radioterapia Robótica, Centro Oncológico Costarricense, San José, CRI

**Keywords:** radiosurgery, meningioma, dura mater, dura tail

## Abstract

Introduction

Meningiomas are extra-axial central nervous system tumors. Complete resection is often curative with macroscopically complete removal of the tumor, excision of its dural attachment, and any abnormal bone. Radiosurgery is also an option for high-risk patients or in patients with surgically residual disease. Dural tail is a typical radiological sign on contrast-enhanced MRI; it can contain tumor cells or be a reaction due to vascular congestion and edema. Radiosurgical planning treatment varies regarding the identification and coverage of the dural tail. This study aimed to retrospectively analyze a series of 143 patients with WHO Grade I meningiomas treated with different radiosurgical platforms, and dosing parameters focused on planning and dose delivery to the dural tail.

Methods

From February 2011 to July 2020, 143 patients with histologically confirmed or radiologically assumed WHO Grade I meningiomas were treated using rotating gamma-ray Infini™ (Gamma [MASEP Medical Science Technology Development Co., Shenzhen, China]), TomoTherapy® (Tomo [Accuray Inc., Sunnyvale, CA]), and CyberKnife® (CK [Accuray Inc.]). All plans were retrospectively reviewed to establish the maximum distance (MaxDis) from the prescription dose to the end of the dural tail and the minimum dose at the dural tail (MinDoseT) at this point. We also established the midpoint distance (MPDis) from the prescription dose to MaxDis and the dose at this point (MPDose). Plans were further distinguished when the physician intended to cover the dural tail versus when not. Patients and tumor response were assessed by imaging and clinical and phone call evaluations.

Results

Of the 143 patients, 81 were treated using Gamma, 34 using Tomo, and 28 using CK. Eighty patients were eligible for follow-up, of whom 58 (72.5%) had an unmistakable dural tail sign. Median follow-up was 1,118 days (range 189-3,496), mean age was 54.5 (range 19-90), and 61 were women, and 19 were men. Overall tumor volume was 6.5 cc (range 0.2-59); mean tumor volumes by different platforms were 2.4, 9.45, and 8 cc; dose prescribed and mean tumor coverage were 14 Gy and 92%, 14.5 Gy and 95%, and 14 Gy and 95.75% with Gamma, Tomo, and CK, respectively. The dural tail was drawn and planned with an attempt to treat in 18 patients (31%); the mean MaxDis, MinDoseT, MPDis, and MPDose were 9.0 mm, 2 Gy, 4.5 mm, and 10.6 Gy, respectively. At last follow-up, tumor control was achieved in 96% of patients for the whole series, and there were no statistical variations regarding tumor volume, dose, conformality, or control when stereotactic radiosurgery was used to cover the dural tail versus when it was not (p=0.105). One patient experienced a Grade 4 Radiation Therapy Oncology Group toxicity as an adverse radiation effect that required surgery, and 11 (7.6%) experienced a Grade 1 toxicity.

Conclusions

This is our preliminary report regarding the efficacy of radiosurgery for meningiomas using diverse platforms at three years of follow-up; the results regarding tumor control are in accordance with the published literature as of this writing. A conscious pursuit of the dural tail with the prescription dose has not proven to provide better tumor control than not doing so - even small areas of the tumor uncovered by the prescription dose did not alter tumor control at current follow-up. The doses delivered to these uncovered areas are quite significant; further follow-up is necessary to validate these findings.

## Introduction

Intracranial meningiomas represent 25% to 38% of primary tumors and are the most common intracranial tumors in the United States [1,2]. Most of them are benign and could be identified in asymptomatic patients as incidental findings. The treatment options are expectant, resective surgery, stereotactic radiosurgery (SRS), or fractionated radiation therapy. SRS has been effective in the long-term control of World Health Organization (WHO) Grade I meningiomas [3,4]. Therefore, SRS is a proper alternative for the initial treatment or as a complementary therapy for residual tumor or recurrence after resection surgery [4-6]. SRS’s effectiveness in controlling this tumor has been reported at least as equivalent with the surgical outcome cataloged as a Simpson Grading System 1 that is characterized when a macroscopically complete removal of a tumor, with excision of its dural attachment, and any abnormal bone, can be done for small and medium-sized meningiomas [4,7].

Despite the vast amount of evidence available regarding SRS’s efficacy in intracranial meningiomas, there are some unresolved issues about planning and target volume, such as the importance of including the dural tail with the prescribed dose [4,8-11]. Although the clinical significance of the dural tail remains unclear, it is a radiological sign with established criteria, such as the greatest thickness adjacent to the tumor and tapering away from it, at least three sections showing the dural tail in magnetic resonance imaging (MRI), and enhancement has to be more intense than the tumor itself [12]. The dural tail is not exclusive to meningiomas since other intracranial tumors can also present this sign on MRI. Nevertheless, its presence correlates well with the diagnosis of meningiomas [12-15]. The dural tail in meningiomas has been histologically studied by several investigators who reported that the dural tail contains tumor cells in up to 61% to 100% of the cases and can extend from 2 to 35 mm from the main tumor mass [14,16,17]. In the remaining cases, the dural tail may not contain tumor but rather tissue proliferation and hypervascularity [14]. Only two studies have sought to demonstrate the importance of trying to cover the dural tail with the prescription dose [9,11]. In everyday practice, multiplatform radiation machine settings and other factors such as experience and planner variability can potentially influence radiosurgical plans for such tumors [8]. Other variables may be present depending on the planning done between forward planning (more typical of gamma units) or inverse planning (more typical in linear particle accelerator units such as TomoTherapy® [Tomo] and CyberKnife® [CK]) (Accuray Inc., Sunnyvale, CA) [18-20].

This research was conducted to understand our current tumor control as it pertains to variability regarding the pursuit or not of the dural tail, doses typically received by this structure regardless of whether it was included in the prescription dose, and how this could vary regarding the different technological platforms for radiosurgery used in our centers with the hopes that emerging units such as ours can benefit from the experience accumulated with the results emerging from different planning characteristics.

## Materials and methods

The present study is a retrospective cohort study, conducted from February 2011 to October 2020, of all the meningioma cases treated with radiosurgery in our centers and that were known or believed to be WHO Grade I (Table 1).

**Table 1:** Demographics and clinical characteristics HA, headache

Variable	N (%)
Simple size	143 patients
Age (mean, in years)	54.5 (range: 19-90)
Female	104 (72%)
Anatomical location	
Convexity	26 (18.3%)
Cavernous sinus	18 (12.6%)
Petrus ridge	25 (17.6%)
Foramen magnum	6 (4.2%)
Parasagittal/falx	22 (15.4%)
Sphenoid wing	10 (7%)
Suprasellar	12 (8.4%)
Olfactory groove	11 (7.7%)
Cerebellopontine angle	9 (6.3%)
Intraorbital	3 (2.1%)
Previous surgery	
Yes	31 (21%)
No	112 (79%)
Neurological symptoms before radiosurgery	
No symptoms	17 (11.9%)
Symptomatic	125 (88%)
HA	32 (25.6%)
Visual alterations with or without HA	23 (18.4%)
Vertigo/gait imbalance	15 (12%)
Seizure	14 (11.2%)
Cranial nerve disfunctions	19 (13.3%)
Others	22 (17.6%)
Neurological symptoms at the last follow-up, n = 111	
None	19 (17%)
Improved	61 (55%)
Same	25 (22.5%)
Worse	6 (5.5%)
Tumor volume	6.5 cc (range: 0.2-59.6)
Treatments by radiosurgical platforms	
Gamma	81 (57%)
TomoTherapy	34 (23%)
CyberKnife	28 (19.7%)
Single fraction	124 (87.3%)
Multifraction	18 (12.6%)

Since 2011, we have used Tomo for delivering either single session or multifractional radiosurgery schemes. In 2014, a rotating gamma-ray unit known as Infini™ (Gamma) (MASEP Medical Science Technology Development Co., Shenzhen, China) was also incorporated to treat these tumors. More recently, in February 2018, CK was also used to treat these lesions. For radiosurgical treatment and data collection, informed consent was obtained from all patients. This study was approved by the local Institutional Ethical Committee and Review Board of the International Cancer Center Group in San Salvador, El Salvador.

**Figure 1:**
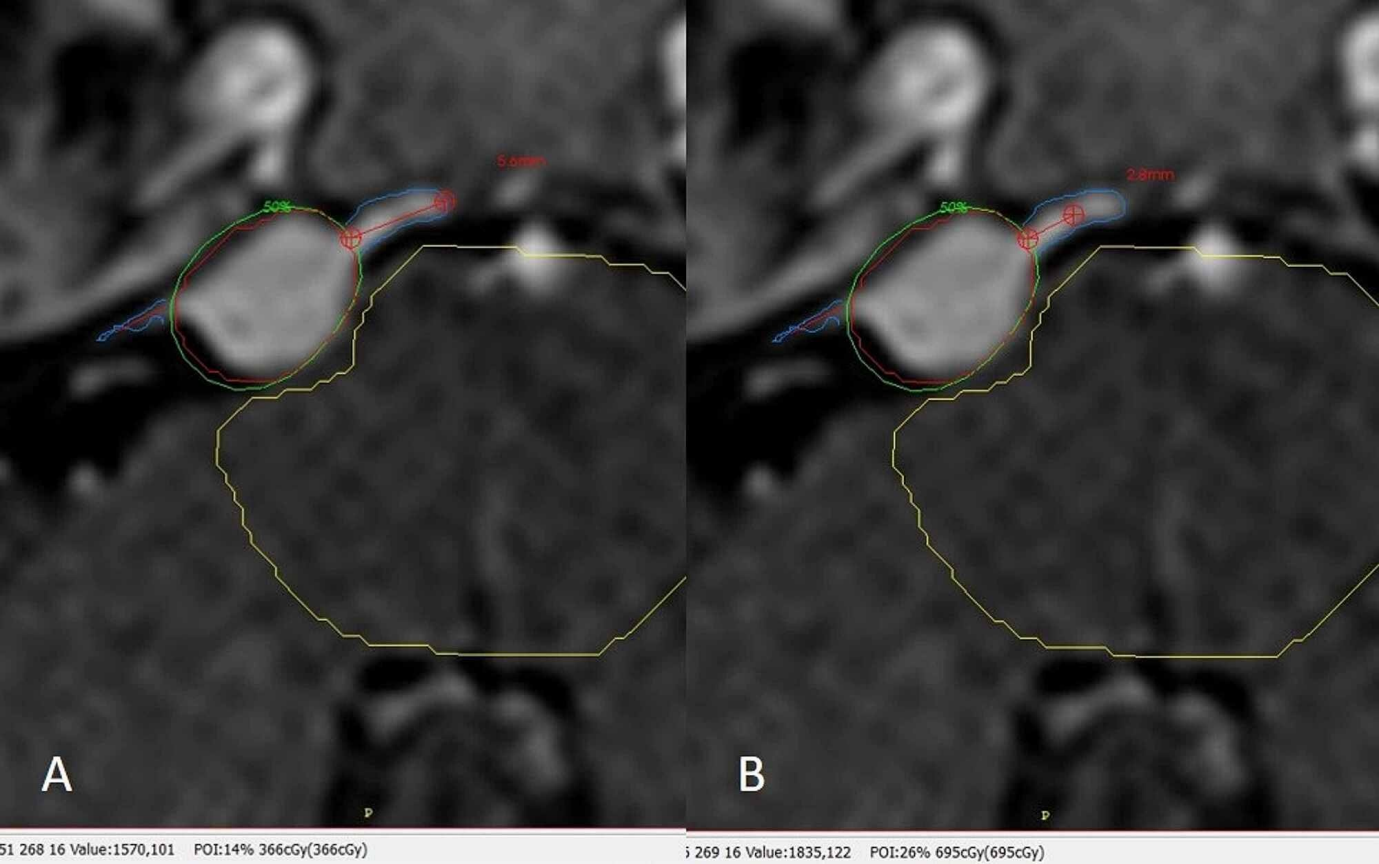
Dural tail identification and measurement process Gad, gadolinium; MaxDis, maximum distance; MinDoseT, minimum dose at the dural tail; MPDis, midpoint distance; MPDose, midpoint dose. Axial T1 Gad where the tumor has been outlined in red (planning target volume), the dural tail sign has been identified to the lateral and medial side of the lesion. The green isodose line covering the tumor is the prescription dose. (A) The red line in the medial tail marks the MaxDis, that in this case is 5.6 mm and the MinDoseT at this point can be seen at the most inferior part of the image in parenthesis corresponding to 3.66 Gy. (B) The red line in the medial tail now marks the MPDis, that is, now half of MaxDis 2.8 mm and the MPDose at this point can also be seen at the most inferior part of the image in parenthesis corresponding to 6.95 Gy.

All plans were reassessed with a particular interest in the identification of the dural tail sign and the final prescription isodose line regarding tumor coverage and measurement of the dose received in the uncovered areas at the maximum distance (MaxDis) of the dural tail and the midpoint distance (MPDis) from the prescription isodose line to the maximum dural tail distance. Regarding the dural tail identification, the inclusion criteria was as follows: the tail had to be visible at least in three consecutive sections of the MRI used for planning (usually a T1-weighted multiplanar gradient recall gadolinium with 1-mm slices), and the greatest thickness needed to be adjacent to the tumor and tapering away from it. Plans were reviewed to establish the MaxDis from the prescription dose to the end of the dural tail and thus, the minimum dose at the dural tail (MinDoseT), and also the MPDis from the prescription dose to MaxDis and the dose at this point (MPDose) that was delivered during treatment (Figure 1).

**Figure 2:**
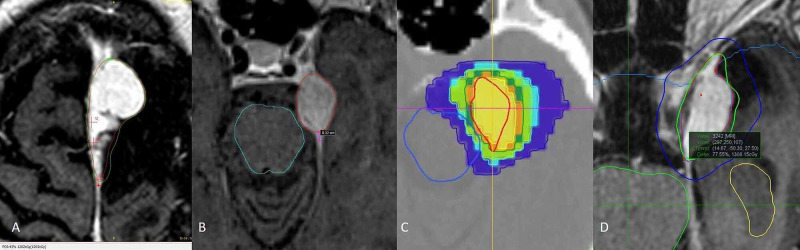
Plans performed with the different treatment systems CK, CyberKnife; Gad, gadolinium; MaxDis, maximum distance; MinDoseT, minimum dose at the dural tail; MPDis, midpoint distance; MPDose, midpoint dose; MRI, magnetic resonance imaging; Tomo, TomoTherapy. (A) Axial T1 Gad showing an interhemispheric meningioma treated with gamma; the green isodose line corresponds to the prescription dose of 14 Gy where an attempt to cover much of the tail is performed; at the most posterior aspect of the tumor there is a red mark signaling the end of the dural tail. The dose at this point can be read at the bottom of the image and corresponds 12.2 Gy at MinDoseT. (B) Contouring on MRI T1 Gad with Tomo of an anterior tentorium, left meningioma outlined in red, posterior to the tumor the measurement line in magenta signals the MPDis that is 3.2 mm. (C) Same meningioma in Tomo’s treatment planning station; prescription dose is 14 Gy, and the dose measurement at MPDose is 14.3 Gy. (D) Axial T1 Gad showing a treatment plan, isodose curves for CK of a left cavernous sinuous meningioma being prescribed 14 Gy represented with the green isodose line; most exterior curve in blue represents dose gradient; the outside measurement dot in the most posterior aspect of the lesion signals the MinDoseT receiving 13 Gy as can be read in the green box at the middle of the image.

At different time points and in accordance with experience, treatment planning modalities by technologies, such as forward (Infini) versus inverse (Tomo, CK) strategies, changed among the main planners. Some stressed the need to cover the dural tail while others would not; thus, plans were further separated when there was a clear intention to pursue the dural tail versus not pursue the tail. Finally, all doses for multifractional schemes were converted to biological equivalent dose (BED) to single-fraction equivalence using an alpha/beta ratio of 4. Multiplatforms provide different plans and dose gradients, and a visual example of each plan regarding Infini, Tomo, and CK and dural tail measurements is provided in Figure 2.

Patients’ symptoms, toxicity, and tumor response were assessed by imaging with a minimum cutoff of six months and clinical or phone call evaluations. Statistical analysis consisted of categorical variables expressed as absolute numbers and percentages. Continuous variables were expressed as median and interquartile range (IQR). Differences among groups were assessed using the chi-square test for categorical variables and analysis of variance or Kruskal-Wallis test as appropriate for continuous variables. p-values<0.05 indicated statistical significance.

**Figure 3:**
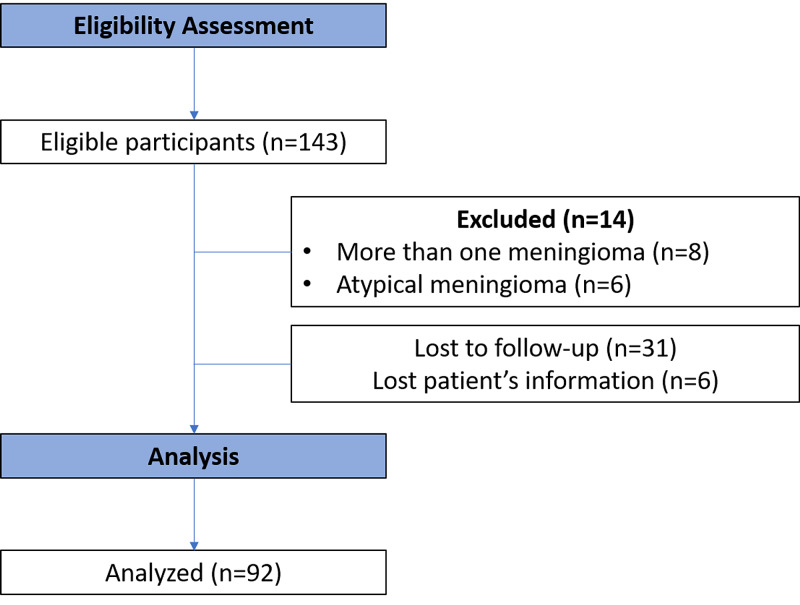
Scoping literature review process

To compare our results and determine the evidence of the dural tail inclusion in the radiosurgery treatment of meningiomas, we carried out a scoping review to identify all original studies in which the dural tail was included in the radiosurgery volume target. From database inception to December 4, 2020, we searched Medline with the following Medical Subject Headings (MeSH) search terms: “radiosurgery” and “meningioma” (Figure 3).

## Results

We identified 143 patients in our database, among whom 81 were treated using Gamma, 34 using Tomo, and 28 using CK. Fourteen patients were excluded as they harbored more than one meningioma (n=8) or further histological analysis confirmed atypical features (n=6), 31 were lost to follow-up, in 6 patients, plans could not be retrieved for analysis, and 12 were excluded due to shorter than six-month follow-up and imaging. Therefore, 80 patients were eligible for follow-up, and 58 (72%) of them had an unmistakable dural tail sign. Median follow-up was 1,118 days (range 189-3,496), mean age was 54.5 (range 19-90); 71 were women and 21 were men. Most patients had some form of neurological symptoms (88%), and 31 (21%) had at least one previous surgery (Table 1). Overall tumor volume was 6.5 cc (range 0.2-59), and mean tumor volumes by different platforms were 2.4, 9.45, and 8 cc. Tumor volumes were statistically different for Gamma as compared to Tomo and CK (p<0.05), but were not different between Tomo and CK (p=0.8). Most treatments were delivered in a single session (n=124; 87.3%), for three- or five-day multifractional schemes, BED to a single dose was 13 Gy (range 12-15). The overall dose prescribed and mean tumor coverage was 14 Gy (range 12-16) and 92% (range 88%-97%), 14.5 Gy (range 12-16) and 95% (range 87%-98%), and 14 Gy (range 13-15) and 95.75% (range 88%-99.7%) with Gamma, Tomo, and CK, respectively.

The dural tail was drawn and planned with an attempt to treat in 18 (31%) patients. Overall, the mean MaxDis, MinDoseT, MPDis, and MPDose were 9.0 mm (range 1.4-23.8), 4.0 Gy (range 0.5-14), 4.5 mm (range 1-16.2), and 10.6 Gy (range 3.8-15). For those tumors where the dural tail was drawn with an attempt to treat versus when it was not, the mean follow-up was 1,186 days (range 212-3,384) versus 1,207 days (range 189-3,496; p=0.641). The MaxDis, MinDoseT, and MPdose were 12.1 Gy (range 6.9-15) versus 10.3 Gy (range 3.8-14), p=0.006, and there was no statistical difference in tumor control between both groups (Table 2).

**Table 2:** Tumor and stereotactic radiosurgery parameters with regard to dural tail coverage for those available to follow-up SRS, stereotactic radiosurgery; DT, dural tail; MaxDis, maximum distance from the prescription dose to the end of the dural tail; MinDoseT, minimum dose at the dural tail; MPDis, midpoint distance from the prescription dose to the maximum distance; MPDose, dose at the midpoint distance; PTV, planning target volume; RECIST, response evaluation criteria in solid tumors. ^a^Patients with SRS delivered to the dural tail vs patients who did not receive SRS to the dural tail, with Kruskall-Wallis test. ^b^Significant difference p<0.05.

Variable	DT SRS	Non-DT SRS	p-value^a^
N (%)	18 (31%)	40 (68.9%)	
Follow-up (days)	1,186 (212-3,384)	1,207 (189-3,496)	0.641
Tumor volume	6.4 cc (0.76-38.9)	5.6 cc (0.5-21.8)	0.574
Dose and distance to the dural tail			
MaxDis	9.8 mm (2.9-23.8)	8.7 mm (1.4-23.9)	0.653
MinDoseT	4.5 Gy (1.7-14)	3.5 Gy (0.5-10.1)	0.076
MPDis	4.7 mm (0.9 11.4)	4.4 mm (0.7-11.2)	0.305
MPDose	12.1 Gy (6.9-15)	10.3 Gy (3.8-14)	0.006^b^
Dose to tumor	14 Gy (13-14)	14 Gy (12-16)	0.051
Tumor (PTV) coverage	92% (85-98.3)	92% (85-97)	0.367
Conformity number	0.8 (0.7-0.9)	0.8 (0.7-0.9)	0.794
RECIST <3	17 (95%)	38 (95.5%)	0.574

There were also no statistical variations in tumor control regarding tumor volume at the last follow-up (Table 3).

**Table 3:** Tumor control regarding tumor volume RECIST, response evaluation criteria in solid tumors; N, number. Pearson’s chi-squared test p-value = 0.574. There was no meaningful statistical difference between the different tumor volumes in comparison with tumor control (p>0.05).

Tumor volume (range)		N (%)	RECIST I	RECIST II	RECIST III	RECIST IV
0.1-5 cc	33 (35.8)		2	2	29	0
5.01-10 cc	27 (29.3)		0	1	25	1
10.01-15 cc	14 (15.2)		0	2	11	1
15.01-20 cc	11 (11.9)		0	3	7	1
Larger than 20 cc	7 (7.6)		0	1	6	0
Total			2	9	78	3

The MaxDis, MinDoseT, MPDis, and MPDose for Gamma were 7.4 mm (range 1.4-14), 2.0 Gy (range 0.1-5.7), 3.1 mm (range 1-10.1), and 10.3 Gy (range 3.8-15.5), respectively. For Tomo, the values were 12 mm (range 4.4-15.1), 9.6 Gy (range 3.1-12), 4.9 mm (range 2-10), and 13.4 Gy (range 6.2-15.7). For CK, the values were 9.12 mm (range 4.7-23.5), 4.4 Gy (range 1.8-10), 4.5 mm (range 2.3-12), and 11.6 Gy (range 9-14). The MinDoseT and MPDose were statistically different between Gamma and CK when compared to Tomo (p=0.0001).

At the last follow-up, the crude radiographic control rate was 96% for the whole series (Figure 3). One patient treated with CK experienced a Grade 4 Radiation Therapy Oncology Group toxicity as an adverse radiation effect that required surgery, and 11 patients experienced a Grade 1 toxicity (10.7%). Six of these patients corresponded to Gamma and three to the Tomo group, and the remaining two corresponded to the CK group. Further analysis of the plan could not be performed at the last follow-up for those with multiple meningiomas. For the whole series including those with less than six months’ follow-up (n=111), 19 (17%) had no neurological symptoms, 25 (22.5%) were the same, 61 (55%) referred improvement in their symptoms, and 6 (5.5%) were worse.

Regarding the scoping review of the literature, we identified 1,500 records. Eleven studies were selected based on their title and abstract, and after full-text evaluation, only two studies remained (Table 4) [9,11].

**Table 4:** Study characteristics of published studies about radiosurgery and DT inclusion GK, Gamma Knife; CK, CyberKnife; DT, dural tail; FU, follow-up; SRS, stereotactic radiosurgery; Tomo, TomoTherapy. *This study reports a significant statistical difference in the tumor control between DT inclusion and non-DT inclusion into the radiosurgery target volume (p=0.038).

Author (year)	Type of study	Sample size (N)	Radiosurgery technology	Total dose (Gy)	Tumor control (%)		Toxicity (%)	FU (years)
					DT SRS	Non-DT SRS		
Bulthuis et al. (2014) [11]	Retrospective	160	GK	11-15	95.1	NR	1.2%	5
DiBiase et al. (2004)* [9]	Retrospective	162	GK	4-25	96	77.9	9.3%	4.5
Current series (2021)	Retrospective	80	Gamma, CK, Tomo	14	95	95.5	10.7%	3.2

## Discussion

This study highlights the relevance of the dural tail’s inclusion in the radiosurgery treatment of WHO Grade I meningioma. This is a relatively large (N=80), single-institution retrospective cohort, with a median overall follow-up of three years; therefore, the results can be considered preliminary for a slow-growing tumor such as this one. We confirmed that radiosurgery with Gamma, Tomo, and CK is effective and safe in controlling most intracranial meningiomas. The crude radiographic control rate was 96%, which agrees with other series using different schemes of radiosurgery [4,21-26] at the same time interval of follow-up, and complications were mainly mild in less than 10% of the series.

Regarding the dural tail as a prognostic variable of the effectiveness of treatment with radiosurgery, this work failed to reflect a difference between delivering a higher dose to this structure (MPDose 12.1 Gy) while attempting to cover more of the dural tail as opposed to when the plan tends to cover just the tumor mass (MPDose 10.3 Gy) delivering a habitual prescribed dose of 14 Gy.

Radiosurgical planning varies from one physician to another as well as from center to center [8,27]. We could evidence this discrepancy in our center and the difference in planning even between the same planner using the same treatment and planning platform. Radiosurgery for WHO Grade I meningioma has proven to be effective in long-term local control in most series [3,4,21,26], but little discussion or evidence exists regarding contouring the gross tumor volume (GTV) and whether it should include what is known as the dural tail or its different portions, despite that it has been demonstrated that a large proportion of this structure might contain tumor cells [14,16-17]. The largest study that has resected dural tails examined histopathological characteristics from convexity meningiomas and consisted of 179 patients [14]. They found that 88.3% of dural tails contained tumor cells within 2.5 cm of the tumor base. Although the study managed to do a surgical resection of meningiomas, it raised the question of whether this extent of the dural tail should be included in the target volume of those patients treated with radiotherapy or radiosurgery and if this inclusion could alter the course of tumoral control rate.

Sorting out the interrelation between tumor size and the dural tail pursuit has become a debatable matter. DiBiase et al. have been the only group to conclude on their series that encompassing the dural tail extension by the prescription isodose line ensures better tumor control (crude radiographic control rate 91%) [9]. On the other hand, another study [10] attributed that pursuing the dural tail may result in larger target volume and raised concerns about provoking larger complication rates without achieving the expected outcomes of improvement with regard to long-term local control [11]. In this last publication, the authors delivered gamma knife radiosurgery only on the part of the dural tail that was closely related to the tumor mass, concluding that the exclusion of the rest of the dural tail from the target volume was not associated with an increased risk of tumor growth; however, we stress that they did not run statistical analysis to determine this conclusion.

The optimal target definition to meningiomas has not been prospectively addressed, and the scarce evidence regarding covering or “pursuing” the dural tail with the prescription dose is contradictory. Therefore, we seek to review our evidence where there was an apparent attempt to do so. There was a tendency over the years to include this portion with the belief that this would result in better tumor control. We were unable to demonstrate a significant difference in tumor control when a higher dose was delivered to the tail. Nevertheless, we advise that this initial follow-up was just three years, and tumor control was high; therefore, conclusions based on our results are initial. There is recent published evidence that out-of-field local failure could take more than seven years to occur and could occur in up to 15% of the cases and that prescription dose higher than 13 Gy proved to be important for local tumor control [21]. These recent data stress the need for further follow-up of the current series to understand if our failure to demonstrate a difference regarding dural tail coverage holds true over time. Bulthuis et al. found that excluding the dural tail from the main tumor target did not lead to higher out-of-field tumor progression at the five-year follow-up, even when using a lower prescription and marginal doses than the ones reported here [11]. Based on the out-of-field long-term failure rate in recently published series with longer follow-up [21], more contemporary evidence of peripheral tumor infiltration, improved or optimal target definition possibly with other diagnostic modalities such as positron emission tomography/computed tomography, and thus, future dosing recommendations to the tumor borders need to be constructed. Despite the shortcomings of the present investigation regarding radiosurgery to the dural tail, the study remains beneficial as it has established the basic information regarding dose and distance from the main tumor mass in the dural tail when different planning strategies are conducted.

In our series, tumor control seemed unaffected even to other variables such as tumor volume that has been known to be a predictor of tumor control failure [4,26], possibly reinforcing the fact that there is still a short follow-up interval or that larger tumor volumes were treated using Tomo and CK using fractionated stereotactic radiotherapy with dose schemes providing a BED as close to 14 Gy instead of sacrificing dose with single-fraction regiments [4].

Lastly, conducting a retrospective review of every plan showed us that the quality of forward planning was clearly influenced by experience and possibly by other factors such as knowing that the patient had a head frame placed and awaited treatment and that multiple plans were difficult or impossible to evaluate to determine the best possible plan with the current planning station capabilities. Pursuing the tail requires more complexity in the plan and might require more “shots”, resulting in longer treatment times. When the dose to the tail was analyzed in the current investigation, it became clear that at least half of the dural tail received a significant amount of dose above 10 Gy, even though it was not included in the GTV. The current dose threshold and amount of inclusion of the dural tail sign for out-of-field long-term progression-free survival is unknown. Gamma and CK produce significantly better outcomes than Tomo [27], and as reflected in the current investigation, the dose at the end and middle of the dural tail was significantly higher for Tomo than the other two platforms. This indicates that Tomo’s dose gradient index is significantly lower than the Gamma and CK, and thus, our group no longer uses Tomo in our radiosurgical treatment of these lesions. However, we note that the patients treated with Tomo had the longest follow-up, and their tumor control and toxicity rate were not different from Gamma and CK.

## Conclusions

Although the dural tail sign is frequently seen in meningiomas, its clinical importance for radiosurgery planning remains unclear. Based on our series report with a mean follow-up of three years, and in accordance with the current literature, we conclude that the inclusion of dural tail in the radiosurgery target volume is not associated with better results in terms of tumor control regardless of the type of technology, tumor volume, or anatomical location. However, given the possibility that the dural tail might be infiltrated with tumor cells, the short follow-up of the current series and the lack of levels of evidence of published studies, randomized controlled trials with quality assurance for contouring and dosimetry and further follow-up are advisable.
